# Supplementary material for: Diagnostic performance of serum metabolites biomarker associated with colorectal adenoma: a systematic review
Source: PeerJ. 2024 Sep 20;12:e18043. doi: 10.7717/peerj.18043 (PMC11418823; doi:10.7717/peerj.18043)
Supplement: Table S1 [file peerj-12-18043-s002.docx]

**Supplementary Table 1: QUADOMICS assessment of included studies**

| ITEM | **Zhu et al., (2014)** | **Farshidfar et al. (2016)** | **Long et al. (2017)** | **Chen et al., (2017)** | **Uchiyama et al. (2017)** | **Liu et al. (2018)** | **Gu et al. (2019)** | **Tevini et al. (2022)** | **Guo et al. (2023)** | YES  % | NO  % | Unclear  % |
| --- | --- | --- | --- | --- | --- | --- | --- | --- | --- | --- | --- | --- |
| 1. Were selection criteria clearly described? | Y | Y | Y | Y | Y | Y | Y | Y | Y | 100 | 0 | 0 |
| 2. Was the spectrum of patients representative of patients who will receive the test in practice? | Y | Y | Y | Y | Y | Y | Y | Y | Y | 100 | 0 | 0 |
| 3. Was the type of sample fully described? | Y | Y | Y | Y | Y | Y | Y | Y | Y | 100 | 0 | 0 |
| 4. Were the procedures and timing of biological sample collection with respect to clinical factors described with enough detail? | Y | Y | Y | Y | Y | Y | Y | Y | Y | 100 | 0 | 0 |
| 5. Were handling and pre-analytical procedures reported in sufficient detail and similar for the whole sample? | Y | Y | Y | Y | Y | Y | Y | Y | Y | 100 | 0 | 0 |
| 6. Is the time period between the reference standard and the index test short enough to reasonably guarantee that the target condition did not change between the two tests? | ? | ? | Y | Y | Y | Y | Y | ? | Y | 77 | 0 | 33 |
| 7. Is the reference standard likely to correctly classify the target condition? | Y | Y | Y | Y | Y | Y | Y | Y | Y | 100 | 0 | 0 |
| 8. Did the whole sample or a random selection of the sample receive verification using a reference standard of diagnosis? | Y | Y | Y | Y | Y | Y | Y | Y | Y | 100 | 0 | 0 |
| 9. Did patients receive the same reference standard regardless of the result of the index test? | Y | Y | Y | Y | Y | Y | Y | Y | Y | 100 | 0 | 0 |
| 10. Was the execution of the index test described in sufficient detail to permit replication of the test? | ? | ? | Y | Y | Y | Y | Y | Y | Y | 88 | 0 | 22 |
| 11. Was the execution of the reference standard described in sufficient detail to permit its replication? | ? | ? | Y | Y | Y | Y | Y | Y | Y | 88 | 0 | 22 |
| 12. Were the index test results interpreted without knowledge of the results of the reference standard? | N | N | N | N | N | N | N | N | N | 0 | 100 | 0 |
| 13. Were the reference standard results interpreted without knowledge of the results of the index test? | Y | Y | Y | Y | Y | Y | Y | Y | Y | 100 | 0 | 0 |
| 14. Were the same clinical data available when test results were interpreted as would be available when the test is used in practice? | N | N | N | N | N | N | N | N | N | 0 | 100 | 0 |
| 15. Were interpretable / intermediate test results reported? | N | N | N | N | N | N | N | N | N | 0 | 100 | 0 |
| 16. Is it likely that the presence of over-fitting was avoided? | ? | Y | Y | ? | N | Y | Y | ? | Y | 56 | 11 | 33 |

Y=criteria achieved, N=criteria not achieved, ?=Unclear
